# *QuickStats:* Prevalence of Complete Tooth Loss* Among Adults Aged ≥65 Years,^†^ by Federal Poverty Level^§^ — National Health and Nutrition Examination Survey, United States, 1999–2018

**DOI:** 10.15585/mmwr.mm6937a8

**Published:** 2020-09-18

**Figure:**
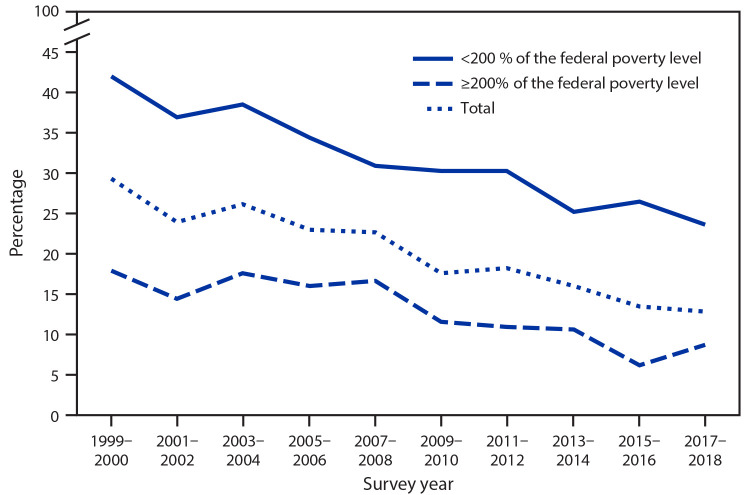
The age-adjusted prevalence of complete tooth loss among adults aged ≥65 years decreased from 29.3% during 1999–2000 to 12.6% during 2017–2018. For the same period, the prevalence decreased from 42.1% to 23.5% for adults living at <200% of the federal poverty level and from 17.7% to 8.5% for adults living at ≥200% of the federal poverty level. Throughout the period, the prevalence of complete tooth loss was higher among those living at <200% of the federal poverty level.

**Sources**: Fleming E, Afful J, Griffin SO. Prevalence of tooth loss among older adults: United States, 2015–2018. NCHS data brief, no. 368. https://www.cdc.gov/nchs/products/databriefs/db368.htm. National Center for Health Statistics, National Health and Nutrition Examination Survey, 2015–2018. https://www.cdc.gov/nchs/nhanes.htm.

